# Supplementary figures and images for: A two–tiered system for selective receptor and transporter protein degradation
Source: PLoS Genet. 2022 Oct 10;18(10):e1010446. doi: 10.1371/journal.pgen.1010446 (PMC9584418; doi:10.1371/journal.pgen.1010446)

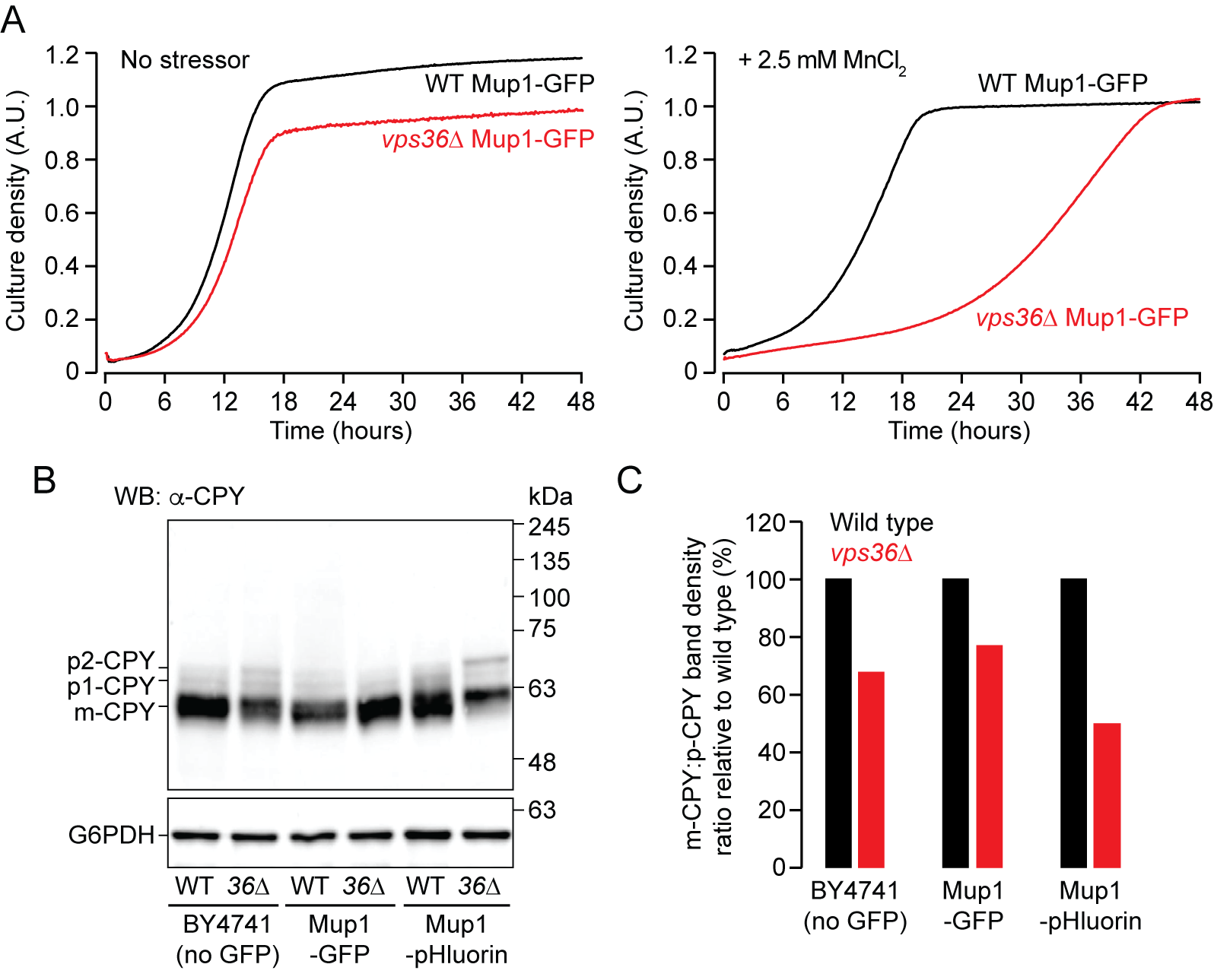

Supplement: S1 Fig — (A) Wild type or vps36Δ cells expressing Mup1-GFP were grown in the absence (no stressor) or presence of 2.5 mM MnCl2 and culture density was recorded over time. Traces shown are representations of 5 independent experiments. Growth of vps36Δ cells is sensitive to MnCl2 as expected. (B) Western blot analysis of whole cell lysates prepared from wild type (WT) or vps36Δ cells expressing no GFP (BY 4741), Mup1-GFP or Mup1-pHluorin. Blots were stained for carboxypeptidase Y (CPY) or glucose-6-phosphate dehydrogenase (G6PDH; as load controls). Smaller mature (cleaved, m-CPY) and larger precursor forms of the enzyme (p1-CPY, p2-CPY), and estimated molecular weights are shown. (C) Mean steady–state m-CPY:p-CPY band density ratios were calculated, normalized to corresponding G6PDH band densities, and are shown for vps36Δ relative to isogenic wild type (WT) for each strain genotype shown in B. n = 3 for each strain tested. vps36Δ strains tested show relatively low amounts of m-CPY as expected. (TIF) [file pgen.1010446.s001.tif]
