# Supplementary material for: A two–tiered system for selective receptor and transporter protein degradation
Source: PLoS Genet. 2022 Oct 10;18(10):e1010446. doi: 10.1371/journal.pgen.1010446 (PMC9584418; doi:10.1371/journal.pgen.1010446)
Supplement: Attachment — Submitted filename: Gloden_et_al-2022-PLoSGen-R2R.pdf [file pgen.1010446.s007.pdf]

RESPONSE TO REVIEWERS

We thank the reviewers for insightful and constructive comments. We addressed all of them to improve the study and manuscript.

Reviewer #1:
In this study the Brett lab continues to reveal the intricacies of the ILF degradation pathway. Specifically, they show that the ILF pathway can serve as a second level of degradation for proteins that normally go through the ESCRT-dependent multvesicular body path. They followed the trafficking of the methionine transporter Mup1 in wild type cells and those with deletions in the in the ESCRT machinery. Mup1 is internalized upon the addition of Met and is trafficked to the vacuole as intraluminal vesicle cargo of multivesicular bodies. Upon fusion Mup1 is directly dilieverd to the lumen of the vacuole where it is degraded. When ESCRT was disabled Mup1 trafficked to the limiting membrane of the vacuole and was subsequently sorted to the boundary domain of docked vesicles. When vacuoles fuse together the Mup1 in the boundarly domain was interanalized as an ILF and degraded. This clearly shows that the ILF pathway is not only independent of the ESCRT machinery, but can also serve as a backup to ensure that membrane proteins are degraded when no longer needed.

This study was executed very well with the proper controls and their conclusions are supported by the data. I believe this paper will be of high impact in the field and suggest that it published as is.

Thank you. We appreciate the concise summary and positive comments.

Reviewer #2:
I'd like to start by apologizing to the authors for the delay in my review of this manuscript; I completely understand the stress of having to wait beyond the suggested timeframe for receipt of a review. Unfortunately, COVID does not appear to respect the peer review process. Please accept my apology, and thank you for your patience and understanding.

In this manuscript by Golden, et al., the authors present a series of interesting in vivo and in vitro experiments to provide insight into the relevance of the yeast vacuolar intralumenal fragment (ILF) pathway in the degradation of unfolded polytopic proteins. While much previous research has shown that turnover of these proteins is usually ESCRT complex-dependent, yeast strains lacking ESCRT complex activity remain able to degrade some membrane proteins through the ILF pathway, especially when grown in the presence of stressors that impact protein stability (like heat). Interestingly, this ESCRT-independent pathway appears to be selective (only half of the proteins tested appear to be degraded in this manner), and the authors suggest that the ILF pathway is a 'fail-safe' mechanism designed to clear unfolded/unneeded proteins under conditions where ESCRT proteins cannot complete their functions in MVB/intralumenal vesicle formation. Importantly, the ILF pathway is a result – and potential side effect – of homotypic vacuole fusion, so the machineries which drive vacuole:vacuole fusion should be absolutely required for ILF-dependent protein degradation.

The Brett laboratory is the leader in the dissection of the ILF pathway in yeast, and has already provided several important studies on ILF function. This submitted work relies heavily on HILO microscopy of purified vacuoles and yeast cells to show the effects of ILF on selected cargo proteins (usually Mup1-GFP), and results appear to show that Mup1-GFP is both recruited to vacuole membranes, and then degraded, in the absence of Vps36p, which would confirm the existence of an ESCRT-independent PM/VM protein turnover pathway. Over the past few years, however, a controversy regarding the importance and relevance of ILF in yeast has arisen, and this current study should be also be viewed in the context of that published work. While this manuscript is extremely well-written and straightforward, a number of missing experimental controls and the failure to discuss this current work in the context of recent publications reduce my enthusiasm for the conclusions of this submitted manuscript. If properly addressed, however, this study could provide important new insight into the presence of an alternative protein degradation pathway that does not rely on ESCRT protein activity.

Major Points

Figures 1 and 2: The authors show that turnover of Mup1-GFP, in the presence of methionine, is not ESCRT-dependent (in a vps36Δ) background. This is evidence that ILF is turning over Mup1-GFP under these conditions, which should require homotypic vacuole fusion. The authors should include important homotypic vacuole fusion mutant strains to confirm that rounds of vacuole fusion in vivo are the driving force behind Mup1-GFP turnover. The prediction would be that Mup1-GFP accumulates on VM membranes in vps36Δ vam3Δ strains, but would unable to be turned over due to the lack of vacuole:vacuole fusion. While the microscopy may be problematic due to fragmented vacuoles in these strains, the Western blot analysis of protein turnover (Fig. 2B) should be very doable. If I'm not mistaken, nyv1Δ strains do have unfragmented, large vacuoles, which may be sufficient for this microscopy, although that might also impact MVB:vacuole fusion that could complicate the analysis. All in vivo experiments that show protein turnover via ILF via Western should include a vacuole fusion mutant control strain.

Our model proposes that the ILF pathway degrades Mup1 when ESCRTs are absent and we offer extensive evidence to support it in the original manuscript. We do not hypothesize what occurs when both pathways are blocked. But based on our model there are two potential outcomes: Mup1 is no longer degraded, or another, unknown pathway compensates for the loss of both mechanisms.

To address this concern, we conducted this experiment and present new results in Figure 3F of the revised manuscript. We find that deleting the vacuolar Qa-SNARE VAM3 alone (*vam3*Δ) or with VPS36 (*vps36*Δ*vam3*Δ) prevented Mup1-GFP cleavage assessed by Western blot analysis. Of note, Mup1-GFP from *vps36*Δ*vam3*Δ migrates relatively slowly compared to other strains, perhaps because protein trafficking is severely disrupted in these mutants (although the basis is unresolved). Also, it was very difficult to generate the double mutant, which is why submitting the revised manuscript was delayed. After the third attempt to generate this strain, I suspected that the genetic mutations were synthetically lethal. But the last attempt proved fruitful, although the resulting double mutant grows poorly. Micrographic analysis (i.e. presence of fragmented

vacuoles) and genome sequencing confirmed the mutations were valid. We added the following text to the revised manuscript to describe these new results:

Page 9, line 5: “To further implicate membrane fusion (a requisite for ILF formation) in Mup1 degradation, we deleted VAM3 which encodes the Qa-SNARE required for vacuole fusion (Karim et al., 2018a). If the ILF pathway contributes, we hypothesize that its loss will block Mup1 degradation in *vps36Δvam3Δ* cells. As predicted, cleavage of GFP from Mup1 was abolished in *vps36Δvam3Δ* cells by Western blot analysis (Figure 3F). Of note, Mup1-GFP from double mutant cell lysates migrated slower, consistent with severe protein trafficking defects. Cells missing only VAM3 also showed diminished cleavage. Together, these results suggest that vacuole membrane fusion is important for Mup1 degradation particularly in the absence of ESCRTs.”

In Figure 2C, the authors show that Fth1p is strongly accumulated at the vacuolar boundary membrane, and therefore should be turned over via ILF. The Brett laboratory has already shown that they observe this fact (McNally, 2017). However, recent published work has shown that Fth1-GFP (and other protein cargo) is NOT turned over post-heat stress in different yeast strain backgrounds (SEY vs BY) over at least 4h, even when intralumenal fragments were observed to form (Yang, et al. 2021. JCB). As heat shock is a critical stress used in this submitted manuscript to show protein turnover via ILF, these discrepancies are important to address. In fact, the authors have completely ignored the presence of this 2021 paper, which strongly questions the relevance of the ILF pathway in protein turnover.

Excellent point. We deliberately omitted discussion of this paper from the manuscript for many reasons. For example, we initially submitted this manuscript in early 2020 and it was reviewed three times for publication at two journals, all prior to the publication of Yang et al., 2021. As such, our experimental design does not incorporate strategies to address potential discrepancies. Rather than add a few experiments to an existing and complete study, we reasoned that a better approach is to conduct an entirely new study that directly and comprehensively addresses all issues raised by Ming Li and colleagues. This study is near completion and will be submitted soon as a separate manuscript.

It is important to note that the focus of this study is to understand how proteins are degraded in the absence of ESCRTs, when (ESCRT-mediated) microautophagy is disengaged, eliminating its potential contribution to Mup1 degradation. We also cite studies conducted by many different, independent groups that support of our model and validate our observations.

However, we agree that the 2021 paper should be acknowledged and to address this concern, we added the following text, limiting discussion to potential discrepancies resolved by data presented in the manuscript:

Page 17, line 21: “A recent report challenges whether ubiquitylated proteins are degraded by the ILF pathway, arguing that ESCRT–mediated microautophagy is exclusively responsible instead (Yang et al., 2021). We do not directly test this hypothesis in our study, nor do we present data suggesting ubiquitin is required for protein degradation by the ILF pathway – although it is the focus of ongoing research. However, our results show that when triggered by methionine,

cycloheximide or heat stress, GFP– or pHluorin– tagged Mup1 is degraded within the lumen of vacuoles in cells missing ESCRT genes VPS36 or VPS27 (Figures 2 and 4). Because intact ESCRTs mediate this form of microautophagy (Yang et al., 2021), it unlikely contributes to Mup1 degradation in these mutant cells. In the near real time videos presented (Figures 3D and 4E; Videos 1–3), a vacuole membrane structure resembling intermediates of both pathways are observed within live cells: A lumenally protruding tube or flap connected to the outside membrane on one end and severed on the other side is a structure formed after partial Rab– and SNARE–dependent *fusion* of *two vacuoles* immediately prior to ILF formation (Mattie et al., 2017; McNally et al. 2017; Karim et al., 2018b). A similar structure, called a macroautophagic tubule, is formed after invagination of the membrane lining *a single vacuole*, and subsequent membrane *fission* by ESCRT-III generates lumenal vesicles (Zhu et al., 2017; Yang et al., 2021). Prior to observing these intermediates in these videos, two tethered vacuoles are present and an intact interface containing Mup1–GFP between them is observed spanning the entire contact site, demonstrating that resulting lumenal structures (intermediates and ILFs) are most likely products of membrane fusion. In support, deleting VAM3 a vacuole Qa-SNARE required for fusion in *vps36*Δ cells blocks Mup1 degradation in vivo. Blocking vacuole fusion in vitro with the Rab inhibitor Gdi1 also inhibits Mup1 degradation, confirming that vacuole fusion is necessary. When considering additional evidence presented using complementary approaches, we conclude that the ILF pathway mediates protein degradation in ESCRT mutants."

We also added Yang et al. 2021 JCB to the References in the revised manuscript.

The authors show that a variety of ESCRT mutant strains are not particularly sensitive to a transient heat shock, unlike ssa2Δ or hsa82Δ strains, as measured by methylene blue staining (Fig. 4H,I). The authors conclude that the unfolded proteins in the cell can still be turned over via ILF in these ESCRT mutant backgrounds, thereby reducing the stress of the accumulation of unfolded proteins. If ILF is indeed a 'fail-safe' pathway to remove unfolded proteins, this experiment should be repeated with vacuole fusion mutant strains to show that cell death strongly increases in the escrtΔ fusionΔ strains after heat shock.

We conducted the proposed experiment and results are shown in Figure 4H and I in the revised manuscript. As predicted, we find that *vps36*Δ*vam3*Δ cells are more sensitive to heat stress than *vps36*Δ cells. We added the following text to the revised manuscript to describe these new results:

Page 10, line 34: "Cells lacking VAM3, a Qa-SNARE needed for vacuole fusion and ILF formation, showed sensitivity to heat stress, and cells missing genes needed for both pathways (*vps36*Δ*vam3*Δ) showed greater sensitivity to heat stress (Figure 4H and I), suggesting that Vam3-mediated fusion is likely needed to degrade toxic misfolded proteins in cells, especially those lacking ESCRT function."

In Figure 5, the authors rely on purified yeast vacuoles to show that Mup1-GFP accumulates in the lumen of yeast vacuoles isolated from vps27Δ strains when they are allowed to fuse. The experiment in 5A (non-heat shock) should include fusion inhibitors to show that this accumulation is fusion-dependent.

Good point. In Figure 5G, we show that accumulation of Mup1-GFP in the vacuole lumen after fusion is significantly diminished in the presence of the fusion inhibitor Gdi1 (although only a single time point was analyzed). We also provide evidence for this using an equivalent (arguably more quantitative) assay in Figure 5F. Here we demonstrate that loss of Mup1-pHluorin fluorescence over time, presumably by exposure to low lumenal pH within vacuoles after internalization of Mup1 upon fusion, is blocked by the inhibitor Gdi1. We show that Mup1-GFP cleavage is blocked by Gdi1 (Figure 5H, bottom blot) and Mup1-GFP sorting into boundaries is blocked by Gdi1 (Figure 5D and E). We argue that together these results provide sufficient evidence to support our conclusion that blocking fusion prevents sorting (Figure 5D,E), internalization (5F, G) and degradation (5H) of Mup1-GFP within the lumen of vacuoles.

Fusion inhibitors are used in Fig. 5D (good), but a heat stress on isolated vacuoles seems problematic here. The authors do not include information on using a vacuolar protease inhibitor during in vitro vacuole fusion experiments (Pbi2p), which is potentially problematic. Loss of vacuolar lumenal contents during in vitro fusion reactions does occur at some background level (Starai, et al. 2007. PNAS), which I might expect to increase after a heat shock. Is the degradation of Mup1-GFP observed in this study a result of lysed and resealed vacuoles? Is released and activated Prb1p (or other vacuolar proteases) responsible for this observation?.

Excellent points. Yes we use Pbi2p in our experiments and have clarified this in the text (Page 22, line 34).

Although it is possible that some lumenal proteases escape from isolated vacuoles during heat stress, it is unlikely that this accounts for observe cleavage of GFP from Mup1 because there is no Mup1–GFP cleavage during heat stress in the presence of the fusion inhibitor Gdi1 (Figure 5H). Under these conditions, proteases could leak out and cleave GFP from Mup1 on the outside surface of VMs but the data refutes this possibility.

What happens to Mup1-pHlourin fluorescence when vacuoles isolated from the vps27Δ strains are forced to fuse in the absence of ATP? Supplementation of these reactions with recombinant Vam7p will force vacuole fusion, but will not acidify the lumen of the vacuole

Good point. We have extensively characterized effects of adding recombinant Vam7 on ILF formation during homotypic vacuole fusion (see McNally et al. 2017 Dev Cell; Mattie et al. 2017 MBoC). In sum, using rVam7 to drive fusion prevents selective cargo sorting into boundaries (all proteins get in) and causes abnormally large ILFs to form. Thus, adding rVam7 to prevent lumenal acidification during fusion will introduce confounding effects making results difficult to interpret (noting that rVam7–mediated fusion also enhances acid hydrolase leakage according to Starai et al., 2017 PNAS). In McNally et al. 2017 Dev Cell, we first describe this pHluorin–based assay in detail and provide many controls similar to the requested experiment. For example, to collapse the pH gradient across VMs we use the protonophore nigericin and show that changes in pHluorin florescence are lost (e.g. Supplemental Figure 1 in McNally et al. 2017 Dev Cell). Herein, we show that rGdi1 blocks changes in pHluorin fluorescence as a control and argue that it is sufficient (along with supporting data generated using many other approaches) to support our conclusion.

Overall, the authors have failed to address the Yang, et al. JCB paper that shows that many previously-discovered ILF cargo proteins (Fth1, Cot1, Vph1, etc) fail to be degraded in the absence of ESCRT complex activity, even under CHX or heat shock conditions. This is a major discrepancy that must be addressed in in this manuscript prior to publication;…

We understand and appreciate the concern and it was largely addressed above.

We did not examine Fth1, Cot1 or Vph1 in this study. As they are not the focus of this work, we argue that this manuscript is not the forum to present a detailed discussion to directly address potentially conflicting datasets presented in our other published papers. But as mentioned above, we agree that this is important and are currently working it.

…it is not immediately clear to me why HILO microscopy would be a more sensitive technique for these types of studies, as opposed to microfluidic/confocal real time techniques used in the Yang paper.

We do not argue that real time HILO microscopy is more sensitive technique for studying the ILF pathway. Results from this method (HILO) are adequate to support our conclusions. If it matters, we have previously used real time confocal microscopy (without microfluidics) to unequivocally show that ILFs are produced by two fusing vacuoles in vitro (McNally et al., 2017 Dev Cell). Herein, results from real time HILO microscopy (i.e. movies) show that Mup1-GFP on vacuole membranes within living cells is present in boundary membranes and is internalized within ILFs during homotypic fusion, defining features of the ILF pathway. How Mup1 is delivered to vacuole membranes may be better studied using microfluidics, but we cannot envision how it would further improve visualization of the ILF pathway and homotypic vacuole fusion itself (which in our opinion was not adequately addressed in Yang et al., 2021).

Thus, without extensive comparative analysis, it is currently unclear how the imaging method used could be responsible for potential discrepancies. Based on this, and because we conducted nearly all experiments prior to publication of Yang et al. 2021 in JCB, we cannot justify using their methods (i.e. design and build a custom microfluidics device and acquire an expense confocal microscope with similar imaging capabilities as our existing system) to repeat experiments described in the manuscript.

Does the accumulation of PM proteins on the VM in escrtΔ strains after heat shock depend upon MVB:vacuole fusion? Is this reduced in pep12Δ strains? Getting these PM proteins to the vacuole in the absence of ubiquitination, endocytosis, and delivery to the VM is somewhat mysterious in this background (as the authors note).

Based on the canonical model of the MVB pathway, our impression is that ESCRTs recognize ubiquitylated Mup1 at endosome/MVB membranes after it is labeled by independent ubiquitylation machinery (e.g. E3 ubiquitin ligases, adapter proteins) at the PM and subsequently undergoes endocytosis. In support, we find that Mup1 seems to be ubiquitylated and clearly undergoes endocytosis as predicted because components of ESCRT-I or ESCRT-II are not responsible. ESCRTs do not directly mediate membrane fusion either. Multiple groups including our own have shown that MVB-vacuole membrane fusion persists when components of ESCRTs

are deleted in support of the canonical model (e.g. Karim & Brett 2018 in MBoC). Consistent with these observations, other groups observed the presence of cargo proteins (e.g. Ste3) on VMs in ESCRTΔ cells prior to this study, which motivated us to conduct these experiments as stated in the Introduction (Page 5, Line 7).

Thus, we did not intend to give the impression that Mup1 appearing on vacuole membranes in cells missing ESCRTs is mysterious. We addressed this at length in the Discussion under the subheading "*How are client proteins recognized by both pathways?*" in the original manuscript. However, to address this concern, we modified the text to prevent readers from getting the impression that some observations were "mysterious".

We agree that deleting PEP12 should diminish MVB–vacuole fusion but it is unclear how conducting this experiment would further support our central hypothesis: The ILF pathway can only recognize cargos present on the VM. In ESCRTΔ cells, Mup1 is present on VMs where it is be degraded by the ILF pathway. Although we appreciate the suggestion, we argue that further uncovering the mechanisms that deliver Mup1 to the VM beyond what is already present in the manuscript (e.g. endocytosis and VM delivery of Mup1 is observed in living ESCRTΔ cells; e.g. Figure 1) will not strengthen the central conclusion.

Minor point

Figures 2 and 3 have their final panel (F and E, respectively) in a really strange spot and not in order.

Thank you for bringing this to our attention. We rearranged the panels in these figures to better present the data in the revised manuscript.
