# Supplementary material for: A two–tiered system for selective receptor and transporter protein degradation
Source: PLoS Genet. 2022 Oct 10;18(10):e1010446. doi: 10.1371/journal.pgen.1010446 (PMC9584418; doi:10.1371/journal.pgen.1010446)
Supplement: Attachment — Submitted filename: Golden review.docx [file pgen.1010446.s006.docx]

I’d like to start by apologizing to the authors for the delay in my review of this manuscript; I completely understand the stress of having to wait beyond the suggested timeframe for receipt of a review. Unfortunately, COVID does not appear to respect the peer review process. Please accept my apology, and thank you for your patience and understanding.

In this manuscript by Golden, *et al*., the authors present a series of interesting in vivo and in vitro experiments to provide insight into the relevance of the yeast vacuolar intralumenal fragment (ILF) pathway in the degradation of unfolded polytopic proteins. While much previous research has shown that turnover of these proteins is usually ESCRT complex-dependent, yeast strains lacking ESCRT complex activity remain able to degrade some membrane proteins through the ILF pathway, especially when grown in the presence of stressors that impact protein stability (like heat). Interestingly, this ESCRT-independent pathway appears to be selective (only half of the proteins tested appear to be degraded in this manner), and the authors suggest that the ILF pathway is a ‘fail-safe’ mechanism designed to clear unfolded/unneeded proteins under conditions where ESCRT proteins cannot complete their functions in MVB/intralumenal vesicle formation. Importantly, the ILF pathway is a result – and potential side effect – of homotypic vacuole fusion, so the machineries which drive vacuole:vacuole fusion should be absolutely required for ILF-dependent protein degradation.

The Brett laboratory is the leader in the dissection of the ILF pathway in yeast, and has already provided several important studies on ILF function. This submitted work relies heavily on HILO microscopy of purified vacuoles and yeast cells to show the effects of ILF on selected cargo proteins (usually Mup1-GFP), and results appear to show that Mup1-GFP is both recruited to vacuole membranes, and then degraded, in the absence of Vps36p, which would confirm the existence of an ESCRT-independent PM/VM protein turnover pathway. Over the past few years, however, a controversy regarding the importance and relevance of ILF in yeast has arisen, and this current study should be also be viewed in the context of that published work. While this manuscript is extremely well-written and straightforward, a number of missing experimental controls and the failure to discuss this current work in the context of recent publications reduce my enthusiasm for the conclusions of this submitted manuscript. If properly addressed, however, this study could provide important new insight into the presence of an alternative protein degradation pathway that does not rely on ESCRT protein activity.

**Major Points**

1. Figures 1 and 2: The authors show that turnover of Mup1-GFP, in the presence of methionine, is not ESCRT-dependent (in a *vps36∆*) background. This is evidence that ILF is turning over Mup1-GFP under these conditions, which should require homotypic vacuole fusion. The authors should include important homotypic vacuole fusion mutant strains to confirm that rounds of vacuole fusion in vivo are the driving force behind Mup1-GFP turnover. The prediction would be that Mup1-GFP accumulates on VM membranes in *vps36∆ vam3∆* strains, but would unable to be turned over due to the lack of vacuole:vacuole fusion. While the microscopy may be problematic due to fragmented vacuoles in these strains, the Western blot analysis of protein turnover (Fig. 2B) should be very doable. If I’m not mistaken, *nyv1∆* strains do have unfragmented, large vacuoles, which may be sufficient for this microscopy, although that might also impact MVB:vacuole fusion that could complicate the analysis. All in vivo experiments that show protein turnover via ILF via Western should include a vacuole fusion mutant control strain.
2. In Figure 2C, the authors show that Fth1p is strongly accumulated at the vacuolar boundary membrane, and therefore should be turned over via ILF. The Brett laboratory has already shown that they observe this fact (McNally, 2017). However, recent published work has shown that Fth1-GFP (and other protein cargo) is NOT turned over post-heat stress in different yeast strain backgrounds (SEY vs BY) over at least 4h, even when intralumenal fragments were observed to form (Yang, et al. 2021. JCB). As heat shock is a critical stress used in this submitted manuscript to show protein turnover via ILF, these discrepancies are important to address. In fact, the authors have completely ignored the presence of this 2021 paper, which strongly questions the relevance of the ILF pathway in protein turnover.
3. The authors show that a variety of ESCRT mutant strains are not particularly sensitive to a transient heat shock, unlike *ssa2∆* or *hsa82∆* strains, as measured by methylene blue staining (Fig. 4H,I). The authors conclude that the unfolded proteins in the cell can still be turned over via ILF in these ESCRT mutant backgrounds, thereby reducing the stress of the accumulation of unfolded proteins. If ILF is indeed a ‘fail-safe’ pathway to remove unfolded proteins, this experiment should be repeated with vacuole fusion mutant strains to show that cell death strongly increases in the *escrt∆ fusion∆* strains after heat shock.
4. In Figure 5, the authors rely on purified yeast vacuoles to show that Mup1-GFP accumulates in the lumen of yeast vacuoles isolated from *vps27∆* strains when they are allowed to fuse. The experiment in 5A (non-heat shock) should include fusion inhibitors to show that this accumulation is fusion-dependent. Fusion inhibitors are used in Fig. 5D (good), but a heat stress on isolated vacuoles seems problematic here. The authors do not include information on using a vacuolar protease inhibitor during in vitro vacuole fusion experiments (Pbi2p), which is potentially problematic. Loss of vacuolar lumenal contents during in vitro fusion reactions does occur at some background level (Starai, et al. 2007. PNAS), which I might expect to increase after a heat shock. Is the degradation of Mup1-GFP observed in this study a result of lysed and resealed vacuoles? Is released and activated Prb1p (or other vacuolar proteases) responsible for this observation? What happens to Mup1-pHlourin fluorescence when vacuoles isolated from the *vps27∆* strains are forced to fuse in the absence of ATP? Supplementation of these reactions with recombinant Vam7p will force vacuole fusion, but will not acidify the lumen of the vacuole.
5. Overall, the authors have failed to address the Yang, et al. JCB paper that shows that many previously-discovered ILF cargo proteins (Fth1, Cot1, Vph1, etc) fail to be degraded in the absence of ESCRT complex activity, even under CHX or heat shock conditions. This is a major discrepancy that must be addressed in in this manuscript prior to publication; it is not immediately clear to me why HILO microscopy would be a more sensitive technique for these types of studies, as opposed to microfluidic/confocal real time techniques used in the Yang paper.
6. Does the accumulation of PM proteins on the VM in *escrt∆* strains after heat shock depend upon MVB:vacuole fusion? Is this reduced in *pep12∆* strains? Getting these PM proteins to the vacuole in the absence of ubiquitination, endocytosis, and delivery to the VM is somewhat mysterious in this background (as the authors note).

**Minor point**

1. Figures 2 and 3 have their final panel (F and E, respectively) in a really strange spot and not in order.
